# Mechanism of acetaldehyde-induced deactivation of microbial lipases

**DOI:** 10.1186/1471-2091-12-10

**Published:** 2011-02-22

**Authors:** Benjamin Franken, Thorsten Eggert, Karl E Jaeger, Martina Pohl

**Affiliations:** 1 Institute of Molecular Enzyme Technology, Heinrich-Heine University Düsseldorf, Forschungszentrum Jülich GmbH, D-52426 Jülich, Germany; 2 evocatal GmbH, Merowinger Platz 1a, D-40225 Düsseldorf, Germany; 3 QIAGEN GmbH, QIAGEN Straße 1, D-40724, Germany; 4 Institute of Bio- and Geosciences, IBG-1: Biotechnology, Forschungszentrum Jülich GmbH, D-52425 Jülich, Germany

## Abstract

**Background:**

Microbial lipases represent the most important class of biocatalysts used for a wealth of applications in organic synthesis. An often applied reaction is the lipase-catalyzed transesterification of vinyl esters and alcohols resulting in the formation of acetaldehyde which is known to deactivate microbial lipases, presumably by structural changes caused by initial Schiff-base formation at solvent accessible lysine residues. Previous studies showed that several lipases were sensitive toward acetaldehyde deactivation whereas others were insensitive; however, a general explanation of the acetaldehyde-induced inactivation mechanism is missing.

**Results:**

Based on five microbial lipases from *Candida rugosa*, *Rhizopus oryzae*, *Pseudomonas fluorescens *and *Bacillus subtilis *we demonstrate that the protonation state of lysine *ε*-amino groups is decisive for their sensitivity toward acetaldehyde. Analysis of the diverse modification products of *Bacillus subtilis *lipases in the presence of acetaldehyde revealed several stable products such as *α,β*-unsaturated polyenals, which result from base and/or amino acid catalyzed aldol condensation of acetaldehyde. Our studies indicate that these products induce the formation of stable Michael-adducts at solvent-accessible amino acids and thus lead to enzyme deactivation. Further, our results indicate Schiff-base formation with acetaldehyde to be involved in crosslinking of lipase molecules.

**Conclusions:**

Differences in stability observed with various commercially available microbial lipases most probably result from different purification procedures carried out by the respective manufacturers. We observed that the pH of the buffer used prior to lyophilization of the enzyme sample is of utmost importance. The mechanism of acetaldehyde-induced deactivation of microbial lipases involves the generation of *α,β*-unsaturated polyenals from acetaldehyde which subsequently form stable Michael-adducts with the enzymes. Lyophilization of the enzymes from buffer at pH 6.0 can provide an easy and effective way to stabilize lipases toward inactivation by acetaldehyde.

## Background

Microbial lipases (triacylglycerol hydrolases, EC 3.1.1.3) belong to the *α/β*-hydrolase family of enzymes [1,2] and catalyze the hydrolysis of triglycerides to glycerol and fatty acids [3,4]. Furthermore, lipases are the most frequently used biocatalysts in organic chemistry due to its ready availability and low cost production, lack of cofactors, broad substrate specificity and high enantioselectivity as well as high stability in non-aqueous media such as ionic liquids, supercritical fluids and organic solvents [5,6]. Under non-aqueous reaction conditions lipases catalyze the synthesis of esters by esterification, inter-esterification, and transesterification [4,7,8].

Lipase catalyzed transesterifications of alcohols with esters are equilibrium reactions that need to be shifted toward the product site. Therefore, several activated esters including enol- and ethoxyvinyl esters have been tested as substrates [9,10] with enol esters such as isopropenyl acetate or vinyl acetate being the most useful acyl donors [11]. After cleavage, the enol tautomerizes to a carbonyl compound (acetaldehyde or acetone) which can no longer serve as a substrate for the back reaction, thus rendering the system fast and irreversible and also facilitating downstream processing due to the volatility of the carbonyl product [9,11]. Furthermore, the stoichiometrically generated acetaldehyde can be used in a high-throughput assay to detect transesterification activities of lipases and esterases directly in the organic phase [12]. Despite of these advantages, aldehydes are generally known to act as alkylating reagents by forming Schiff-bases in a Maillard-type reaction, in particular with enzyme lysine *ε*-amino groups. Although Schiff-base formation as such is a reversible reaction, several follow-up reactions finally render this modification irreversible [13]. Hence, acetaldehyde affects various enzymatic properties as shown for different enzymes of biotechnological significance such as lipases, 2-deoxy-D-ribose 5-phosphate aldolase (DERA), and thiamine-diphosphate dependent enzymes, respectively [14-17]. Acetaldehyde treatment of commercial lipase preparations from *Candida rugosa *and *Galactomyces geotrichum *(formerly *Geotrichum candidum*) resulted in reduced activity and enantioselectivity, whereas only moderate effects were observed with lipases from *Pseudomonas *sp. or *Rhizopus oryzae *[18,19]. *C. rugosa *lipase was stabilized by immobilization, either covalently on an epoxy-activated carrier resin or by adsorption on Celite 545 [18,20]. Aldehyde-based deactivation also represents a problem in several lipase-based industrial biotransformations, e.g. in the formation of *trans*-2-methoxycyclohexanol and 3-(4-methoxyphenyl) glycidic acid [21]. Only recently, the activity of *C. rugosa *lipase was significantly increased by immobilisation of the enzyme in crosslinked-enzyme aggregates (CLEAS) together with BSA as a "proteic feeder" [22]. Further, activity has been improved by "pH-tuning" of lipases prior to their use in organic solvents, meaning that the enzyme was lyophilized from a buffer with optimal pH for biocatalysis [23].

Current hypotheses infer that the sensitivity of lipases toward acetaldehyde depends on: (1) the microbial source from which the lipase originates; (2) its molecular weight; (3) the number of lysine residues; (4) their solvent accessibility; (5) a covalent modification of lid-domain residues (for *G. geotrichum *and *C. rugosa *lipases); and (6) the pK_a_-values of the lysine *ε*-amino groups [19,24]. The latter hypothesis assumed that the nucleophilicity of lysine *ε*-amino groups would define their contribution to the deactivation reaction and calculated pK_a_-values were used to predict Schiff-base formations with lysine *ε*-amino groups of higher pK_a_-values and thus increasing acetaldehyde sensitivity of the respective lipase [24]. Comprehensive mutagenesis studies of 2-deoxy-D-ribose 5-phosphate aldolase [15] and prolipase from *R. oryzae *[14] failed to achieve complete stabilisation against aldehyde deactivation and furthermore indicated the involvement of several different sites in these enzymes.

In this study, we demonstrate that the protonation state of lysine *ε*-amino groups in microbial lipases has a significant impact on their sensitivity against acetaldehyde. We also show that *α,β*-unsaturated polyenals derived from aldol condensation of acetaldehyde in the presence of primary amino compounds or NaOH have a major impact on the deactivation process, most probably by forming stable Michael-adducts with lysine and other side chains.

## Results and Discussion

### The protonation state determines the sensitivity of lipases toward acetaldehyde

Schiff-base formation depends on the protonation state of primary amino groups, as only a deprotonated amino group (-NH_2_) can nucleophilically attack the acetaldehyde carbonyl group. Thus, it can be predicted that enzyme deactivation caused by Schiff-base formation should depend on the pH of the respective reaction buffer. If biotransformations are performed in organic solvents with lyophilized or immobilized enzymes, their protonation state is affected by the so-called "pH-memory effect" meaning that lyophilization traps the protonation state of a protein in solution [25,26]. Different commercial enzyme preparations presumably exhibit different protonation states and additionally, stabilizing additives and/or protein impurities are commonly found resulting in heterogeneities which clearly can influence the deactivation behavior of the respective biocatalyst [27-32]. Therefore, we have adjusted the protonation states of lipases from *Candida rugosa *(CRL), *Rhizopus oryzae *(ROL), *Pseudomonas fluorescens *(PFL), and *Bacillus subtilis *lipases (BSL A and B) by lyophilizing them from buffers of pH 6 and pH 10, respectively, being far below or equal to the average pK_a _of the lysine *ε*-amino groups. The freeze-dried enzymes were then treated with acetaldehyde in toluene and the residual activity was determined. We employed toluene as the solvent for our investigation because it is of significant technical importance and is frequently used in technical lipase processes [21]. It is worth to mention that acetaldehyde-treated lipases were not treated with reducing agents such as sodium cyanoborohydride to stabilize Schiff-bases by converting them into stable secondary amines. Hence, the obtained results can be attributed solely to the formation of stable acetaldehyde-protein adducts resulting from Schiff-bases as precursors.

**Figure 1:**
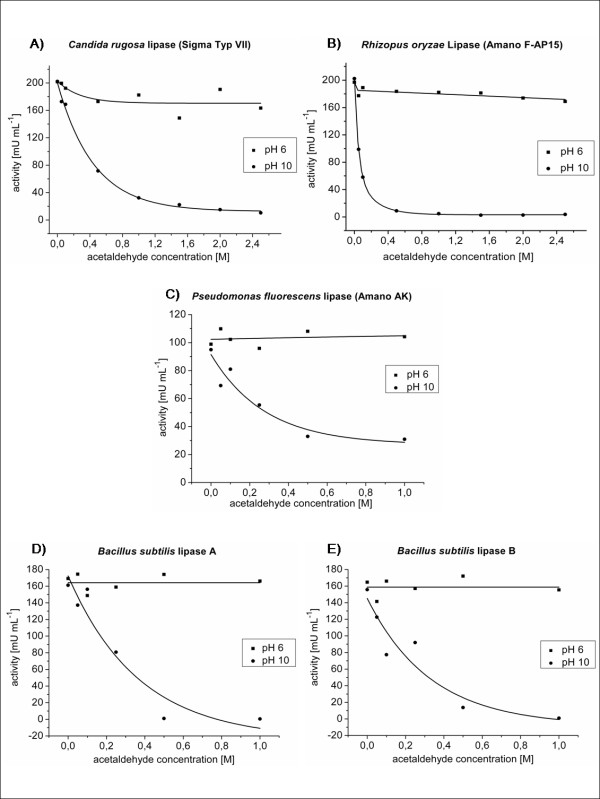
**Deactivation of microbial lipases with acetaldehyde after lyophilization at pH 6 and pH 10, respectively**. A) *R. oryzae *lipase (ROL). B) *C. rugosa *lipase (CRL). C) *P. fluorescens *lipase (PFL). D) *B. subtilis *lipase A (BSL-A). E) *B. subtilis *lipase B (BSL-B). Lipases from *C. rugosa*, *R. oryzae*, and *P. fluorescens *were dissolved in citrate/NaOH-buffer (100 mM, pH 6) or Na_2_CO_3_/NaHCO_3_-buffer (100 mM, pH 10), respectively, at a final concentration of 10 mg/mL. BSL-A and BSL-B were stored in glycine/NaOH-buffer (10 mM, pH 10) and diluted 1:2 with glycine/NaOH (10 mM, pH 10) and citrate/NaOH-buffer (100 mM, pH 6), respectively to a final concentration of 0.5 mg/mL. Samples were incubated in screw-capped vials for 1 h at - 80°C prior to lyophilization carried out overnight at 0.011 mbar. The lyophilized enzymes were treated with toluene, sonicated for 3 min to obtain homogenous dispersions, than incubated with acetaldehyde (0-1 M for BSL-A, BSL-B, and PFL, and 0-2.5 M for CRL and ROL, respectively) and incubated for 24 h at room temperature. Lipases were extracted from the organic phase with 800 μL Na_2_HPO_4_/KH_2_PO_4_-buffer (50 mM, pH 8), and the centrifuged solution was assayed for residual activity using the p-nitrophenylpalmitate assay [63].

**Figure 2:**
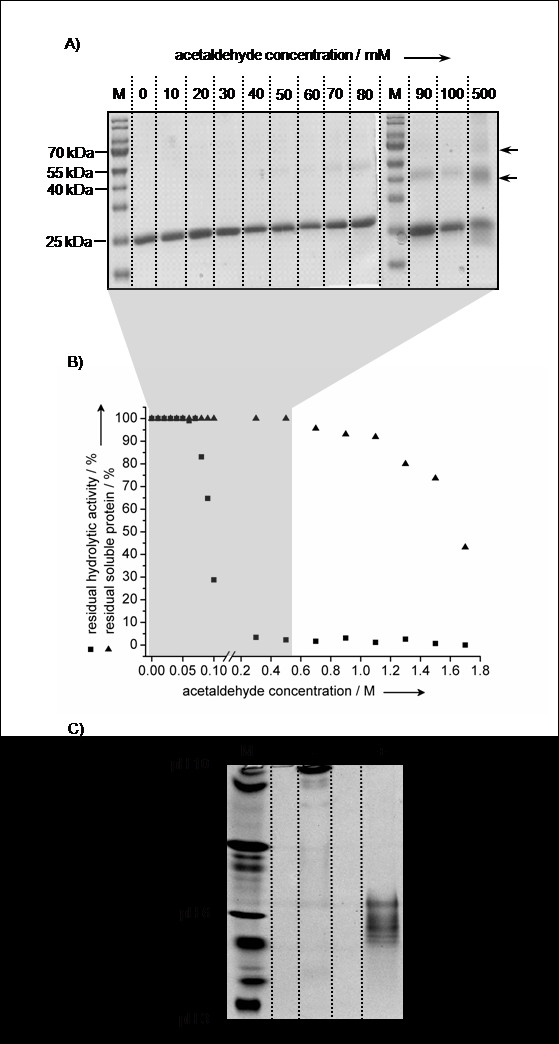
**Acetaldehyde induced modification and inactivation of BSL-B**. A) Sodium dodecyl sulfate polyacrylamide gel electrophoresis (SDS-PAGE) of *B. subtilis *lipase B (BSL-B) treated with acetaldehyde (0-0.5 M) in glycine/NaOH buffer (10 mM, pH 10). S: Molecular weight standard. Arrows indicate putative lipase dimers (~ 40 kDa) and trimers (~ 60 kDa). **B**) Soluble protein concentration and residual hydrolytic activity of BSL-B after treatment for 24 h with acetaldehyde (0-1.8 M) in glycine/NaOH buffer (10 mM, pH 10). **C**) Isoelectric focusing (IEF) gel (pH 3-10) of BSL-B after treatment with (0.5 M) and without acetaldehyde in glycine/NaOH (10 mM, pH 10). M: IEF marker. -: BSL-B without acetaldehyde. +: BSL-B with acetaldehyde.

Three of the studied lipases, namely CRL, ROL, and PFL, were previously investigated, with regard to their acetaldehyde sensitivity [19]. CRL was deactivated strongly (-76%), ROL moderately (-17%), and PFL remained nearly unaffected (-2%) upon overnight incubation with acetaldehyde (0.1 M) in toluene [19]. All enzymes tested showed a significant decrease in activity with increasing acetaldehyde concentrations after lyophilization at pH 10, whereas no (PFL) or only a moderate (ROL and CRL) deactivation occurred in preparations lyophilized at pH 6 (Figure 1). These results demonstrate that even CRL, which was previously classified as highly sensitive against acetaldehyde [19], can be stabilized by lyophilization at a pH far below the average pK_a _of its lysine *ε*-amino groups. These results support findings of Majumder and Gupta [22], who achieved stabilization of CRL. On the other hand, lipases previously described as moderately (ROL) or completely (PFL) stable against acetaldehyde [19] can be rendered sensitive by lyophilization at a pH equal to the pK_a _of their lysine *ε*-amino groups (Figure 1 A-C). The uniform deactivation behaviour of ROL, CRL, and PFL identifies the lipase protonation state including the ratio of NH_2_- to NH_3_^+^- groups as key parameters for acetaldehyde sensitivity. Previous findings suggested a modification of the lid-domain covering the active site as a putative cause of deactivation in *G. geotrichum *and *C. rugosa *lipases [24]. Thus, we compared the deactivation behaviour of BSL-A and BSL-B which share a compact minimal *α*/*β*-hydrolase fold lacking a lid [33,34]. These lipases (Figure 1D+E) show the same deactivation kinetics as CRL, ROL, and PFL (Figure 1A-C) indicating that the modification of the lipase core structure dominates the deactivation behaviour. Additionally, isoelectric focusing experiments revealed a significant shift of the isoelectric point (pI) from about pH 9.5 for the untreated lipase sample toward pH 6 for the acetaldehyde treated sample (Figure 2C). This result suggests that an initial Schiff-base modification may be followed by the formation of stable acetaldehyde-protein adducts. Furthermore, a prediction of the pK_a_-values [35] of solvent accessible lysine residues for all lipases whose structures are solved and deposited in the protein data base (http://www.pdb.org) revealed similar pK_a_-values in all cases, including four of five lipases studied in this work (Additional file 1, Tab. S1). Thus, it can be concluded that acetaldehyde will affect other lipases in a similar manner.

### Acetaldehyde induces cross-links

Formaldehyde-induced modifications of model peptides and insulin demonstrated that a Schiff-base modified lysine residue can form stable intra- and intermolecular cross-links with the side chains of arginine, asparagine, glutamine, histidine, tryptophan, and tyrosine [36,37]. Further, intramolecular cross-links may result from the reaction of lysine-bound Schiff-bases with neighbouring peptide bonds forming 4-imidazolidinone rings [36]. Investigation of possible cross-linked products was performed using BSL-B as a model enzyme. The enzyme was incubated with different concentrations of acetaldehyde (0-0.5 M) in aqueous solution at pH 10 where BSL-B shows maximum activity and stability [38]. Analysis by SDS-PAGE revealed additional bands with higher molecular weight (~ 40 kDa and ~ 60 kDa) appearing at an acetaldehyde concentration of 0.08 M (Figure 2A). The intensity of these bands increased with increasing acetaldehyde concentrations indicating the formation of BSLB-dimers (~ 40 kDa) and trimers (~ 60 kDa) respectively, presumably caused by intermolecular cross-link formation. Parallel determination of the hydrolytic lipase activity and the soluble protein concentration revealed deactivation already at an acetaldehyde concentration of 0.08 M (Figure 2B). Increasing the acetaldehyde concentration to 0.5 M led to a complete deactivation of BSL-B, whereas considerable protein aggregation could be detected only at concentrations above 1.3 M (Figure 2B). Furthermore, a shift toward higher molecular weight was observed for the monomeric form of BSL-B (Figure 2A) which might be explained by a shift of the isoelectric point (pI) due to acetaldehyde-induced modifications. Alves *et al. *[39] demonstrated that the electrophoretic mobility of a highly acidic protein could be increased by modifying acidic amino acid residues, resulting in an increased pI. This change of the pI increases the ability of the protein to bind SDS [39,40]. For BSL-B, acetaldehyde induced modification results in a decrease of the pI (Figure 2C), which in turn probably decreases its ability to bind SDS and thus, explains the observed mobility shift (Figure 2A) [41]. Intramolecular cross-links, which most probably also occur, will potentially not give rise to a defined shift of the electrophoretic mobility, because there a several modification sites present on the protein surface, which prevents the formation of specific crossed-linked products. Separate substitution of each lysine residue in BSL-B against arginine and alanine, respectively, did not result in decreased lipase activity (Additional file 2, Figure S1). Thus, acetaldehyde-induced deactivation is not the consequence of modification of a single "hot-spot" lysine residue as previously suggested for *G. geotrichum *and *C. rugosa *lipases [24].

### Acetaldehyde sensitivity of BSL-B depends on enzyme concentration

**Figure 3:**
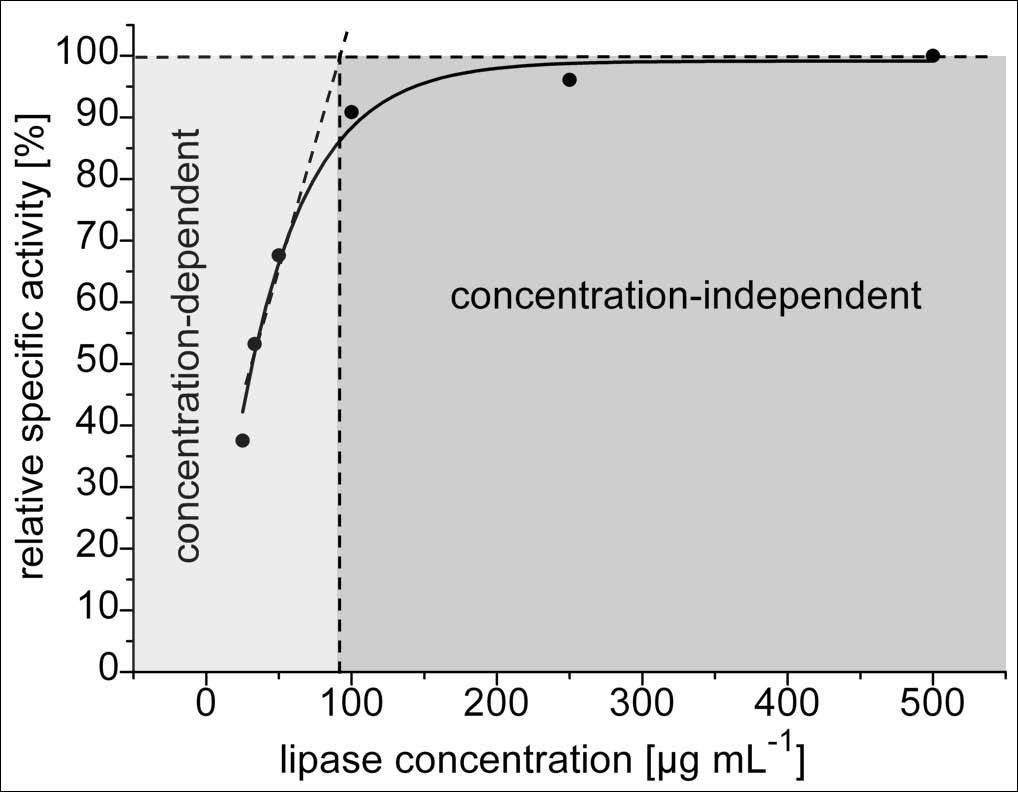
**Acetaldehyde-induced deactivation of BSL-B at different protein concentrations**. Lipase samples were incubated in the presence 500 mM acetaldehyde for 2 h at 37°C and residual enzyme activity determined with *p*-nitrophenylpalmitate as the substrate.

BSL-B samples (0.025 - 0.5 mg/mL in 10 mM glycine/NaOH-buffer, pH 10) were incubated for 2 h in the presence of 500 mM acetaldehyde resulting in a molar excess of at least 2000 acetaldehyde molecules per BSL-B molecule. At higher lipase concentrations no (0.25 and 0.5 mg/mL) or only moderate deactivation (0.1 mg/mL) was observed whereas at lower concentrations (≤0.1 mg/mL) the residual specific lipase activity linearly decreased with decreasing enzyme concentrations (Figure 3).

The deactivation behaviour of BSL-B may be explained by spontaneous aggregate formation, a phenomenon that has already been described for lipases of *C. rugosa *[42], *P. fluorescens *[43], *P. aeruginosa *[44], *Staphylococcus aureus *[45], and BSL-A [46], which is 74% identical to BSL-B [33].

The formation of active and soluble BSL-B aggregates was analyzed by dynamic light scattering over a time range of 24 h (Additional file 3, Tab. S2). Aggregate formation could be detected only at BSL-B concentrations exceeding 0.1 mg/mL. These results indicate that concentration dependent formation of higher molecular lipase aggregates lead to a decelerated deactivation process, probably due to a decreased accessibility of modifiable amino acid side chains within the aggregates.

### Acetaldehyde forms *α,β*-unsaturated polyenals which modify BSL-B

**Figure 4:**
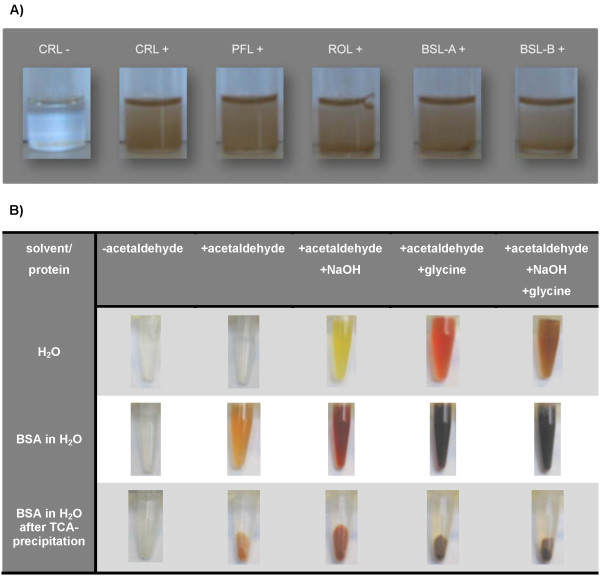
**Acetaldehyde-induced colouration of microbial lipases in toluene (A) and in the presence of different buffer components (B)**. A) Lipases from *C. rugosa *(Sigma type VII, CRL), *P. fluorescens *(Amano AK, PFL), *R. oryzae *(Amano F-AP15, ROL), BSL-A, and BSL-B were lyophilized from buffer adjusted to pH = 10 and incubated in the presence (+) or absence (-) of 500 mM acetaldehyde in toluene. B) Influence of acetaldehyde (500 mM), NaOH (10 mM), and glycine (10 mM) on colour of solutions (upper row), solubilized (middle row) and precipitated (lower row) BSA (1 mg/mL). Therefore, water or BSA in water was incubated with (+) or without (-) acetaldehyde and the buffer components glycine and NaOH. Subsequently, all samples containing BSA were treated with TCA to document the colour of the protein pellet (BSA in water after TCA-precipitation).

Incubation of BSL-B with acetaldehyde in aqueous or non-aqueous solvents (i.e. toluene) resulted in the formation of a yellow to brownish colour (Figure 4A and 4B). The influence of different buffer components on colour formation was analyzed by incubating 500 mM acetaldehyde dissolved in water in the presence of glycine, NaOH and glycine/NaOH, respectively. In all cases, the incubation of acetaldehyde with glycine and/or NaOH led to a yellow to reddish-brown coloured solution (Figure 4B, upper row). The influence of a protein on colour formation was tested with a highly purified preparation of bovine serum albumin (BSA). Upon addition of acetaldehyde, the initially yellow-orange colour shifted to red-brown and finally to black after further addition of glycine and/or NaOH (Figure 4B, middle row). After precipitation with TCA, the protein pellets also were coloured orange to brown (Figure 4B, lower row). Thus, we concluded that (1) in the presence of a base (NaOH) and/or a primary amino compounds (glycine, BSA) acetaldehyde forms coloured compounds; (2) colour formation is intensified by combining a base with primary amino compounds; and (3) the coloured compounds can bind to a protein.

In order to further analyze the coloured reaction products formed in an aqueous solution containing 500 mM acetaldehyde and 20 mM glycine/NaOH-buffer or 10 mM NaOH, respectively, they were extracted from the aqueous solution and subjected to GC/MS analysis, which clearly revealed the presence of *α,β*-unsaturated polyenals in aqueous acetaldehyde/NaOH-samples with 2,4-hexadienal (m/z: 96, 95, 67) and 2,4,6-octatrienal (m/z: 122) being the most prominent compounds in the yellow coloured acetaldehyde/NaOH sample (see upper row of Figure 4B). Our results are supported by earlier findings reported in the literature, which also demonstrated the formation of *α,β*-unsaturated polyenals from acetaldehyde in the presence of bases and primary amino compounds in aqueous solution [47-50]. Specifically, 10 mM glycine in an aqueous 500 mM acetaldehyde solution was shown to catalyze the formation 2,4-hexadienal, 2,4,6-octatrienal, 2,4,6,8-decatetraenal, and longer chained polyenals, which leads to a reddish-brown to black colour [50]. Hence, we assume that the colour formation we have observed is caused by the formation of *α,β*-unsaturated polyenals and the colour differences result from the different sizes of the respective conjugated *π*-electron systems formed.

### *α,β*-unsaturated polyenals bind to BSL-B by Michael addition

In comparison to acetaldehyde, *α,β*-unsaturated polyenals cannot only induce the formation of unstable Schiff-bases with primary amino groups, but are also capable of forming stable Michael-adducts by reaction of their double bond in *α,β*-position with the nucleophilic side chains of arginine, histidine, lysine and serine [51,52]. We analyzed differently composed samples (BSA (1 mg/mL), BSL-B (1 mg/mL), glycine (10 mM), NaOH (10 mM), and acetaldehyde (500 mM) in different combinations) to detect protein-bound carbonyl groups which are formed only by Michael-addition of *α,β*-unsaturated polyenals. The 2,4-dinitrophenylhydrazine (DNPH)-assay revealed the formation of protein-carbonyl compounds only in the presence of NaOH and/or primary amino compounds (glycine or protein). In the presence of glycine, a three- to five-fold higher number of carbonyl groups was detected indicating a high catalytic efficiency of glycine as aldol condensation catalyst. The lower values determined for BSL-B as compared to BSA can be attributed to a lower number of modifiable amino acid residues (60 lysine residues per BSA molecule *versus *10 per BSL-B molecule). A more precise localization of the modification sites was attempted by analysis of acetaldehyde-treated BSL-B with MALDI-TOF-MS. MS-spectra showed considerable heterogeneity and sufficient material for analyses was difficult to obtain due to the modification of predominantly lysine residues which hampered trypsin digestion during sample preparation. Nevertheless, we succeeded to identify a modified peptide fragment with M_w _= 1507.711 Dalton consisting of amino acids Asn_26_-Lys_37_. Michael-addition of crotonaldehyde to the native Asn_26_-Lys_37 _fragment (M_w _= 1437.712 Da) would result in a theoretical molecular mass shift of 70.09 Da to give 1507.802 Da. This result is the first indication that *α,β*-unsaturated polyenals can interact with BSL-B and form stable Michael-adducts with surface exposed amino acid residues. Further indications were obtained by studying the direct inactivation of BSL-B with 2,4-hexadienal (see below), which was among the major *α,β*-unsaturated polyenals identified in our samples.

### 2,4-Hexadienal inactivates BSL-B not only by modification of lysine residues

In order to investigate the direct effect of *α,β*-unsaturated polyenals, we tested the ability of 2,4-hexadienal to inactivate BSL-B at concentrations of 0 mM, 10 mM, 25 mM, and 50 mM. As expected, BSL-B activity decreased with increasing 2,4-hexadienal concentrations. It is interesting to note that incubation of BSL-B with 50 mM 2,4-hexadienal reduced the hydrolytic activity to 19% after 24 h whereas no activity decrease was detected after incubation with the same concentration of acetaldehyde (Figure 2B).

**Figure 5:**
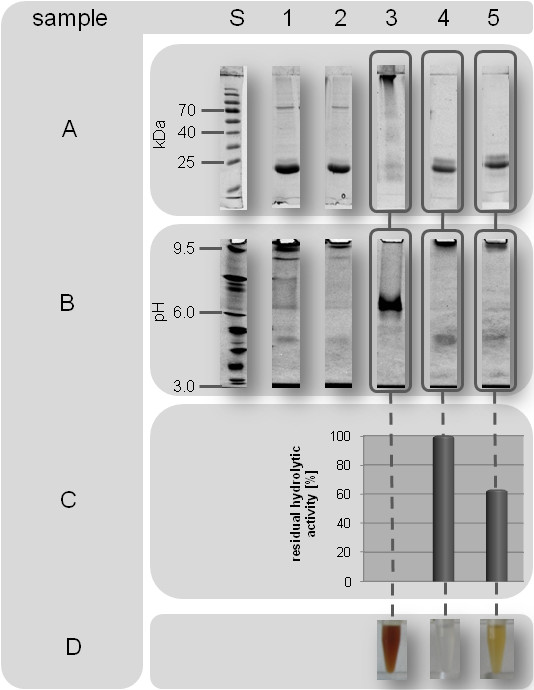
**Acetaldehyde-induced deactivation of BSL-B depends on the number of modifiable amino acid residues and on the presence of primary amino compounds in the solvent**. To investigate the influence protein-bound primary amino groups (N-terminus and ε-amino groups) as well free glycine on the deactivation process, BSL-B (1 mg/mL) has (1) been incubated with (+) or without (-) metylacetimidate; (2) then transferred (+) into fresh glycine/NaOH-buffer (10 mM, pH 10) or not (-); and (3) incubated with (+) or without (-) 500 mM acetaldehyde for 24 h. Subsequently, all samples have been analyzed with SDS-PAGE (A) and IEF (B). Furthermore, the residual hydrolytic activity (C) and the colour (D) of all samples containing acetaldehyde have been documented. S: SDS-PAGE and IEF-Standard. **1**: - methylacetimidate, - glycine/NaOH-buffer, - acetaldehyde. **2**: + methyl-acetimidate, - glycine/NaOH-buffer, -acetaldehyde. **3**: - methylacetimidate, - glycine/NaOH-buffer, + acetaldehyde. **4**: + methyl-acetimidate, - glycine/NaOH-buffer, + acetaldehyde. **5**: + methylacetimidate, + glycine/NaOH-buffer, + acetaldehyde.

To identify the influence of primary amino groups (free glycine, N-termini, and lysine side chains, respectively) on the acetaldehyde induced deactivation, we analyzed differently treated BSL-B samples by SDS-PAGE and IEF, determined their residual hydrolytic activity, and also documented the colour after incubation with 500 mM acetaldehyde (Figure 5). Comprehensive modification of primary amino groups with methylacetimidate (Figure 5, samples 4 and 5, line A and B) abolished the formation of intermolecular cross-links and also a decrease of the pI (compare to Figure 2). These results clearly indicate that both reactions are caused by Schiff-base modified lysine side chains. Subsequently, methylacetimidate was removed and acetaldehyde (samples 3-5) and fresh glycine/NaOH-buffer was added (sample 5). The addition of fresh glycine/NaOH buffer introduced unmodified glycine into the sample and its free amino groups can initiate the formation of α,β-unsaturated polyenals, which was additionally proven by the formation of coloured compounds in samples 3 und 5 (line D). Determination of the hydrolytic activity of samples 4 and 5 demonstrated that the activity of sample 5, which contained α,β-unsaturated polyenals, decreased by ~ 40% relative to sample 4 (defined as 100%). As all solvent accessible amino groups in BSL-B were modified by methylacetimidate, this effect can only be explained by modification of amino acid side chains other than those of lysine (e.g. of arginine, histidine, cysteine) with *α*,*β*-unsaturated polyenals, as has earlier been demonstrated [51,52]. In summary, about 40% of the acetaldehyde induced deactivation of BSL-B can be traced back to Michael-adduct formation at arginine, histidine, and serine side chains (BSL-B possesses no cysteine residues) and about 60% to the Michael-addition of *α,β*-unsaturated polyenals to lysine *ε*-amino groups.

## Conclusions

Lipase-catalyzed transesterification reactions of vinyl esters result in the formation of acetaldehyde which is known to deactivate microbial lipases. Until now, deactivation was thought to be caused by structural changes resulting from initial Schiff-base formation at solvent accessible lysine residues of the enzymes [19,24]. Using BSL-B as a model enzyme we demonstrate that acetaldehyde induced modifications lead to an increase of the enzymes' molecular mass presumably caused by intermolecular cross-linking and a decrease of the isoelectric point, respectively. These stable modifications of the enzyme can be explained by Michael-addition of *α*,*β*-unsaturated polyenals, formed by aldol condensation of acetaldehyde in the presence of bases and/or amino acids [49-52]. Moreover, we found indications that these polyenals act as non-competitive inhibitors of the enzyme.

Our results further demonstrated that acetaldehyde-induced inactivation of microbial lipases can be avoided by adjusting the protonation state of the enzymes. Thus, "pH-tuning" of lipases cannot only preserve activity but also stability of the enzymes by effectively shielding surface exposed amino groups through protonation. This pre-treatment can avoid the formation of *α*,*β*-unsaturated polyenals (if no other bases are present) and protonates primary amino groups which are, besides other nucleophilic side chains, most accessible to Michael-addition. As pH-changes may affect the activity and stability of the enzymes, a prerequisite of pH-tuning is sufficient stability and activity at weak acidic to neutral pH. Besides, free amino acids such as glycine, which is frequently used as a buffer component for lipase preparations, should be avoided for lipase preparations used in transesterification reactions with vinyl esters as they amplify the inactivation process.

**Figure 6:**
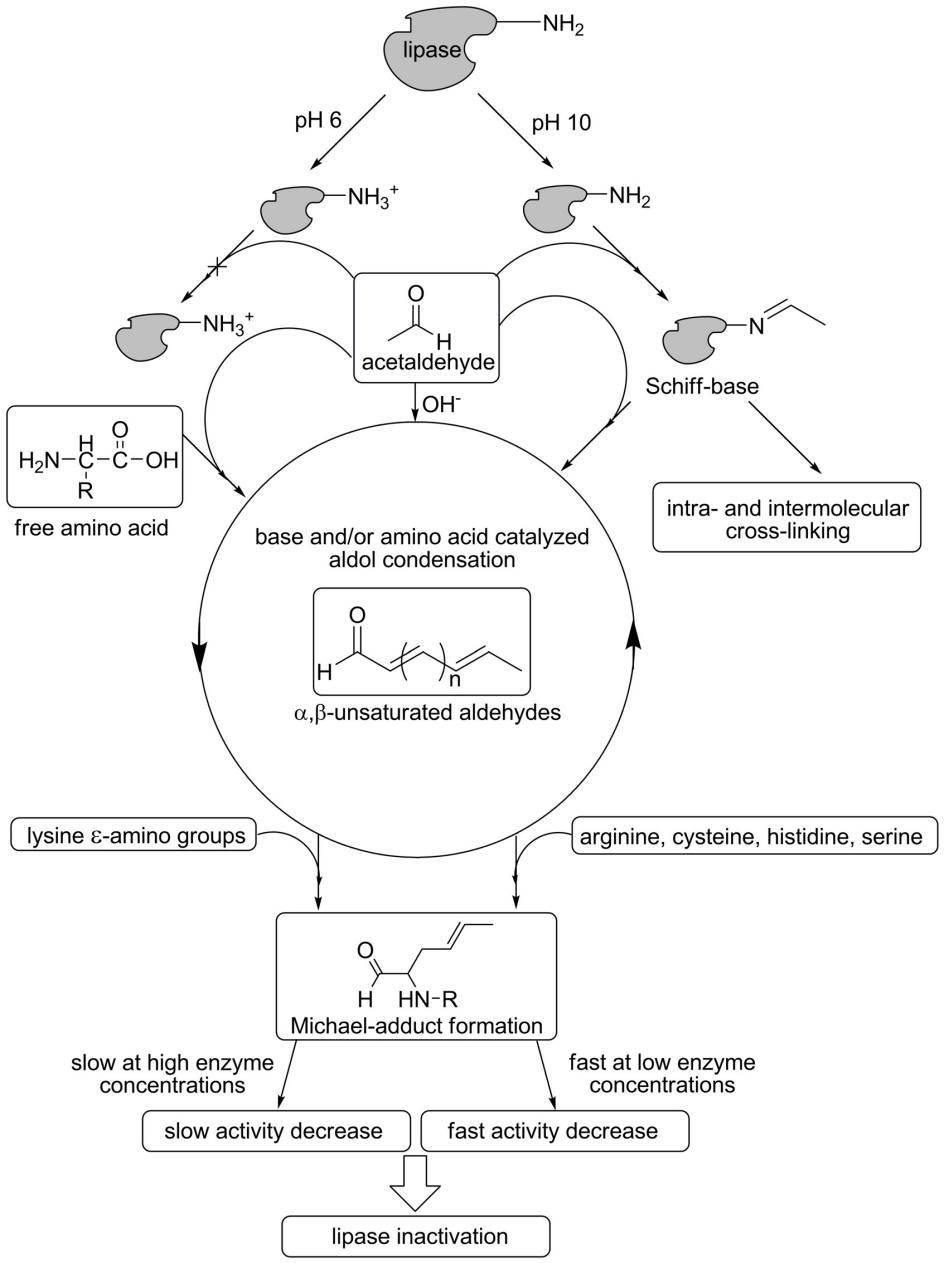
**How acetaldehyde inactivates microbial lipases**. Deprotonated (-NH_2_) or protonated (-NH_3_^+^) amino groups can be generated by lyophilization from buffers of the respective pH. Only deprotonated amino groups are able to form Schiff bases with acetaldehyde; they can induce the formation of intra- and intermolecular cross-links. Furthermore, bases and/or amino compounds (protein-bound amino groups, free amino compounds in the buffer) can catalyze the aldol condensation of acetaldehyde to *α,β*-unsaturated aldehydes which result in a yellow to brownish-black colour of the respective solution or protein. Finally, the formation of Michael-adducts between the enzyme and the *α,β*-unsaturated aldehydes leads to inactivation. At lower enzyme concentrations, the inactivation process proceeds fast; whereas fast aggregation of the enzyme at higher concentrations hampers the covalent modification thereby decelerating the inactivation process.

Our findings also explain the higher stability of several immobilized lipase preparations which is caused by the minimization of solvent-accessible amino groups and other nucleophilic groups. In summary, our results allow us to propose a refined mechanism for acetaldehyde-induced deactivation of microbial lipases as summarized in Figure 6.

## Methods

### Materials

The following chemicals were of analytical grade: acetaldehyde (Fluka, Taufkirchen, Germany), citric acid monohydrate (Carl Roth, Karlsruhe, Germany), glycine (Carl Roth, Karlsruhe, Germany), KH_2_PO_4 _(Carl Roth, Karlsruhe, Germany), sodium dodecyl sulfate (Carl Roth, Karlsruhe, Germany), NaOH (Carl Roth, Karlsruhe, Germany), Na_2_HPO_4 _(Carl Roth, Karlsruhe, Germany), and TCA (Carl Roth, Karlsruhe, Germany).

All other chemicals used in this study were of at least technical grade.

### Enzymes and other proteins

Samples of lyophilized lipases (triacylglycerol hydrolases, EC 3.1.1.3) from *C. rugosa *(CRL, Sigma type VII), *R. oryzae *(ROL, Amano F-AP15), and *Pseudomonas fluorescens *(PFL, Amano AK) were purchased from Sigma-Aldrich (Taufkirchen, Germany). Lyophilized BSA was purchased from Sigma-Aldrich (Taufkirchen, Germany). Lipases A and B from *B. subtilis *(BSL-A and BSL-B) were produced and purified as described previously [53]. Briefly, for large-scale production of BSL-A and BSL-B, high cell density fed-batch cultivation using *E. coli *BL21(DE3) as a host, carrying pET19b derived expression plasmids were performed in an Infors fermenter (30 L) [53-55]. After purification by immobilized-metal affinity chromatography (IMAC) [56] using a Ni-nitrilo-triacetic acid (NTA) superflow column (30 mL, QIAGEN, Hilden, Germany), imidazole was removed by gel filtration chromatography (G-25 column, Amersham Pharmacia Biotech) using glycine/NaOH-buffer (10 mM, pH 10).

### Determination of protein concentrations

Protein concentrations were determined with the Bradford method [57] using bovine serum albumin (BSA) as the standard.

### Adjustment of lipase protonation state

Lipases from *C. rugosa*, *R. oryzae*, and *P. fluorescens *were dissolved in citrate/NaOH-buffer (100 mM, pH 6) or Na_2_CO_3_/NaHCO_3_-buffer (100 mM, pH 10) respectively at a final concentration of 10 mg/mL. BSL-A and BSL-B were stored in glycine/NaOH-buffer (10 mM, pH 10) and diluted 1:2 with glycine/NaOH (10 mM, pH 10) and citrate/NaOH-buffer (100 mM, pH 6), respectively to a final concentration of 0.5 mg/mL. Samples were incubated in screw-capped vials for 1 h at - 80°C prior to lyophilization carried out overnight at 0.011 mbar. In order to avoid pH-shifts due to the change of temperature during freezing, buffers with a small delta pKa/dT were chosen: citrate buffer: dpKa/dT = 0; carbonate buffer: dpKa/dT = -0.009.

### Acetaldehyde-induced deactivation under non-aqueous conditions

The lyophilized enzymes were (1) treated with toluene; (2) sonicated for 3 min (Sonorex ultrasonic bath, BANDELIN electronic, Berlin, Germany) to obtain homogenous dispersions in organic solvent; (3) treated with acetaldehyde (0-1 M for BSL-A, BSL-B, and PFL, and 0-2.5 M for CRL and ROL respectively); and (4) incubated for 24 h at room temperature in an overhead mixer (Reax 2 overhead mixer, Heidolph Instruments, Schwabach, Germany). Lipases were extracted from the organic phase with 800 μL Na_2_HPO_4_/KH_2_PO_4_-buffer (50 mM, pH 8) and the solution subsequently clarified by centrifugation.

### Analysis of aldol products of acetaldehyde in the presence of glycine and NaOH

In order to identify the nature of the coloured products, 500 mM acetaldehyde was incubated in the presence of a 20 mM glycine/NaOH buffer and in 10 mM NaOH without glycine. After incubation at 37°C overnight, the reaction products were extracted with dichloromethane and subjected to GC/MS analysis (Siemens, Varian).

### Treatment of BSL-B with methylacetimidate

BSL-B was treated with methylacetimidate which results in specific modification of primary amino groups which retain their positive charge and still exhibit high stability at alkaline pH [58]. 2 × 1 mL BSL-B solution (1.0 mg/mL in 10 mM glycine/NaOH-buffer, pH 10) were mixed with 1 mL methylacetimidate solution (0.9 M methylacetimidate in 1 M NaOH, pH 9.5) and incubated for 1 h at room temperature as described previously [59]. An untreated sample without methylacetimidate was used as a control. The glycine/NaOH-buffer in one of the BSL-B samples was substituted with fresh glycine/NaOH-buffer (10 mM, pH 10) using a Sartorius Vivaspin-20 centrifugal concentrator column (4°C, 3000 g) and precipitated proteins were removed by centrifugation. Residual hydrolytic lipase activity was determined with *p*-nitrophenylpalmitate as the substrate.

### Acetaldehyde- and 2,4-hexadienal-induced deactivation of BSL-B

BSL-B (0.025-0.5 mg/mL) dissolved in glycine/NaOH-buffer (10 mM, pH 10) was mixed with acetaldehyde (final concentration: 0-1.8 M), or 2,4-hexadienal (final concentration: 0-50 mM), incubated for 2 h or 24 h at 37°C in a thermo mixer (Eppendorf, Germany) at 300 rpm and the solution clarified by centrifugation.

### Acetaldehyde-induced modification of bovine serum albumin

The reaction of acetaldehyde (0-0.5 M) with buffer components and BSA (1 mg/ml) was performed by incubation of samples for 24 h at 37°C in a thermo mixer (Eppendorf, Germany) at 300 rpm and precipitation with trichloroacetic acid (TCA) as described [60] by mixing samples with 1/10 volume of 70% (w/v) TCA, incubation on ice for 30 min and subsequent centrifugation.

### Determination of lipase activity

Lipase indicator plates [61] were prepared by addition of 50% (v/v) tributyrin and 5% (w/v) gum arabic to molten Luria-Bertani agar medium (500 ml) [62]. Esterase and lipase activity is indicated by the formation of clear halos around the colonies of *E. coli *BL21(DE3) expressing an active lipolytic enzyme. Lipase activity in solution was measured with *p*-nitrophenylpalmitate as the substrate as described [63].

### Dinitrophenylhydrazine assay

Protein-bound carbonyl groups were detected as previously described [64] by addition to each sample of 500 μL 2,4-dinitrophenylhydrazine (DNPH) solution (0.1% (w/v) DNPH in 2 M HCl) for 1 h at room temperature with mixing every 15 min (test tube shaker Reax top, Heidolph, Schwabach, Germany). Proteins were precipitated with 500 μL 20% (w/v) TCA by incubation for 15 min on ice and isolated by centrifugation for 30 min. The pellets were washed 3-times with 1 mL 1:1 (v/v) ethyl acetate/ethanol, resuspended in 6 M guanidinium-hydrochloride, 133 mM Tris-HCl buffer containing 13 mM EDTA and the absorption determined at 375 nm.

### QuikChange^®^-PCR

Each lysine residue of BSL-B was replaced by alanine and arginine, respectively, using the QuikChange^® ^method (Stratagene, [65]). Plasmid pET19b carrying the *B. subtilis lip*B gene was amplified using specific mutagenesis oligonucleotide primers (Additional file 4, Tab. S3) and competent *E coli *DH5α and BL21(DE3) cells were transformed with plasmids containing the mutant genes using a standard procedure [66].

### Protein gel electrophoresis

Sodium dodecyl sulfate polyacrylamide gel electrophoresis (SDS-PAGE) was performed using a 5% stacking and a 12% separating gel [67]. Samples of 20 μg BSL-B were loaded per lane and PageRuler™ prestained protein ladder (Fermentas, St. Leon-Rot, Germany) was used as molecular mass standard. Gels were stained as described [68].

### Isoelectric focusing (IEF)

BSL-B samples in glycine/NaOH-buffer (10 mM, pH 10) were treated with acetaldehyde (0.5 M), incubated overnight at 37°C in a thermomixer (Eppendorf, Germany) at 300 rpm, concentrated using a micro-concentrator column (Centricon-3, Amicon, USA) and loaded on a pre-cast vertical IEF gel (Novex^® ^pH 3-10, Invitrogen, Germany) [69]. The SERVA^® ^liquid mix IEF marker (SERVA^® ^electrophoresis, Germany) was used as the pI standard and gels were stained as described [68].

### Dynamic light scattering (DLS)

Aggregation of BSL-B in glycine/NaOH-buffer (10 mM, pH 10) was analyzed in a temperature controlled Protein Solutions™ DynaPro™ dynamic light scattering instrument (Wyatt Technology Corporation, USA). BSL-B samples (0.1-0.5 mg/mL) were incubated for 24 h at 37°C and the hydrodynamic radii and polydispersities were calculated at frequent intervals using Dynamics V6 software (Wyatt Technology Corporation, USA).

### MALDI-TOF-mass spectrometry

BSL-B (0.5 mg/mL) was dissolved in glycine/NaOH-buffer (10 mM, pH 10), incubated with 0 mM, 50 mM, and 500 mM acetaldehyde for 24 h at 37°C in a thermo mixer (Eppendorf, Germany) at 300 rpm, and analyzed by SDS-PAGE. Coomassie-stained protein bands were cut out and gel slices were incubated 2 times for 10 min in 650 μL 0.1 M ammonium bicarbonate in 30% (v/v) acetonitrile, dried for 20 min in a vacuum centrifuge, rehydrated in 6 μL Tris/HCl-buffer (3 mM, pH 8.8) and digested with 10 ng/μL trypsine (Promega) overnight at room temperature. Peptides were isolated by addition of 5 μL A. bidest., incubation for 15 min at room temperature, addition of 5 μL 0.2% (v/v) trifluoroacetic acid in 30% (v/v) acetonitrile and incubation for 10 min at room temperature. For MALDI-TOF-MS-analysis 0.5 μL of the peptide solution were co-crystallized on a steal plate (Bruker Daltonics) with 0.5 μL matrix (α-cyano-4-hydroxy-trans-cinnamic acid in 50% (v/v) acetonitrile and 0.25% (w/v) trifluoro acetic acid). External calibration was performed by using the Peptide Calibration Standard for mass spectrometry (Bruker Daltonics). The samples were analyzed with an Ultraflex MALDI-TOF/TOF mass spectrometer III (Bruker Daltonics) in positive reflector mode at an acceleration potential of 26.3 kV.

### Identification of colored compounds

A solution of 1 mL 10 mM NaOH was incubated with 500 mM acetaldehyde; the coloured compounds were extracted with dichlormethane and analyzed by coupled gas chromatography and mass spectrometry.

### Calculation of pK_a _- and solvent accessibility values

Solvent accessibility and pK_a_-value calculations were performed with the PROPKA web interface (http://propka.ki.ku.dk/~drogers/) [35] using all lipase structure coordinates available at the Brookhaven Protein Database (http://www.pdb.org) and a homology based structural model for BSL-B [33], respectively.

## List of abbreviations used

BSL-A: *Bacillus subtilis *lipase A; BSL-B: *Bacillus subtilis *lipase B; CRL: *Candida rugosa *lipase; DERA: 2-deoxy-D-ribose 5-phosphate aldolase; DLS: dynamic light scattering; DNPH: 2,4-dinitrophenylhydrazine; GC/MS: gas chromatography/mass spectrometry; IEF: isoelectric focusing; MALDI-TOF-MS: matrix assisted laser desorption/ionization time of flight mass spectrometry; PFL: *Pseudomonas fluorescens *lipase; *p*NPP: *para*-nitrophenyl palmitate; ROL: *Rhizopus oryzae *lipase; SDS-PAGE: sodium dodecyl sulfate polyacrylamide gel electrophoresis; TCA: trichloroacetic acid.

## Authors' contributions

This work is the main part of the doctoral thesis of BF, who conceived the study, carried out the experimental part of the work and was mainly responsible for the experimental setup as well as initial drafting of the manuscript. TE and KEJ contributed to this work with their expertise concerning bacterial lipases. Further KEJ helped to draft the manuscript. MP participated in the design and coordination of the study and was mainly involved in the preparation of the manuscript. All authors read and approved the final manuscript.

## Supplementary Material

Additional file 1 **Table S1**: Predicted pK_a_-values of solvent accessible lysine ε-amino groups derived from all available lipase protein structures. Click here for file

Additional file 2 **Figure S1: Tributyrine plate assay of BSL-B wild type and BSL-B point variants**. Tributyrine plate assay of BSL-B wild type as well as BSL-B point variants in which each lysine residue is substituted by alanine and arginine, respectively. -: *E. coli *BL21(DE3) carrying the empty vector pET19b. WT: *E. coli *BL21(DE3) expressing BSL-B wild type enzyme (pET19b + *lip*B). K X A/R: *E. coli *BL21(DE3) expressing BSL-B in which the lysine residue (K) at position X is substituted with alanine (A) or arginine (R). Click here for file

Additional file 3 **Table S2**: Periodically measured DLS data of differently concentrated BSL-B samples. Click here for file

Additional file 4 **Table S3: PCR Primers **QuikChange-PCR-primer sequences for site-directed mutagenesis of each lysine residue in BSL-B for alanine and arginine, respectively. Each primer pair (e.g. *lip*B-K25R-fw und *lip*B-K25A-fw) differs only in the mutagenesis sequence (bold and underlined). Click here for file
