# Supplementary material for: Mechanism of acetaldehyde-induced deactivation of microbial lipases
Source: BMC Biochem. 2011 Feb 22;12:10. doi: 10.1186/1471-2091-12-10 (PMC3049140; doi:10.1186/1471-2091-12-10)
Supplement: Additional file 1 — Table S1: Predicted pKa-values of solvent accessible lysine ε-amino groups derived from all available lipase protein structures. [file 1471-2091-12-10-S1.DOC]

**Additional File 1**

**Mechanism of acetaldehyde-induced deactivation of microbial lipases**

**Benjamin Franken, Thorsten Eggert, Karl E. Jaeger, Martina Pohl**

**Table S1:** Predicted pK_a_-values of solvent accessible lysine ε-amino groups derived from all available lipase protein structures**.**

| **Organism^↓^** | **pdb ID^†^** | **pK_a_-min^‡^** | **pK_a_-max^$^** |
| --- | --- | --- | --- |
| *Bacillus stearothermophilus* | 1ji3  1ku0 | 9.73  9.94 | 10.5  10.5 |
| ***Bacillus subtilis*** | **1i6w**  **1isp***  **1r4z**  **1r50**  **1t2n**  **1t4m** | **9.87**  **9.8**  **9.87**  **9.66**  **9.8**  **9.73** | **10.5**  **10.5**  **10.5**  **10.5**  **10.5**  **10.5** |
| *Bos taurus* | 1akn | 9.59 | 10.5 |
| *Burkholderia cepacia* | 1hqd  1oil  1ys1  1ys2  2lip  3lip  4lip  5lip  1cvl  1qge  1tah  2es4 | 10.29  10.08  10.08  10.08  10.08  10.08  10.01  10.08  10.15  10.15  9.94  10.01 | 10.5  10.43  10.5  10.5  10.5  10.5  10.5  10.5  10.5  10.5  10.5  10.5 |
| *Candida antarctica* | 1lbs  1lbt  1tca  1tcb  1tcc | 9.8  9.8  9.87  9.8  9.73 | 10.5  10.36  10.5  10.43  10.5 |
| ***Candida rugosa*** | **1crl**  **1gz7**  **1llf**  **1lpm**  **1lpn**  **1lpo**  **1lpp**  **1lps**  **1trh** | **9.45**  **9.38**  **9.87**  **9.31**  **9.52**  **9.45**  **9.52**  **9.52**  **9.59** | **10.5**  **10.5**  **10.5**  **10.5**  **10.5**  **10.5**  **10.5**  **10.5**  **10.5** |
| *Canis familiaris* | 1k8q  1rp1 | 9.8  10.08 | 10.5  10.5 |
| *Equus caballus* | 1hpl | 9.8 | 10.5 |
| *Fusarium solani pisi* | 1cua  1cub  1cuc  1cud  1cue  1cuf  1cug  1cuh  1cuj  1cuu  1cuv  1cuw  1cux  1cuy  1cuz  1ffa  1ffb  1ffc  1ffd  1ffe | 9.66  9.73  9.73  9.87  9.66  9.66  9.66  9.73  9.66  9.66  9.8  9.52  9.66  9.66  9.66  9.73  9.73  9.73  9.66  9.59 | 10.5  10.5  10.5  10.5  10.5  10.5  10.5  10.36  10.5  10.5  10.43  10.43  10.5  10.5  10.5  10.5  10.5  10.5  10.5  10.5 |
| *Geobacillus zalihae* | 2dsn  2z5g | 9.94  9.8 | 10.5  10.5 |
| *Geotrichum candidum* | 1thg | 9.66 | 10.5 |
| *Homo sapiens* | 1f6w  1gpl  1hlg  1jmy  1lpa  1lpb  1n8s  2oxe  2ppl | 9.38  9.87  9.94  9.45  9.73  9.94  9.87  9.87  9.8 | 10.5  10.5  10.5  10.5  10.5  10.5  10.5  10.5  10.5 |
| *Penicillium camembertii*^#^ | 1tia | - | - |
| *Pseudomonas aeruginosa* | 1ex9 | 9.73 | 10.5 |
| *Pseudomonas mendocina* | 2fx5 | 10.43 | 10.43 |
| *Pseudomonas sp. MIS38* | 2z8x  2z8z | 10.15  10.15 | 10.5  10.5 |
| *Rattus norvegicus* | 1bu8 | 9.59 | 10.5 |
| *Rhizomucor miehei*^#^ | 1tgl  3tgl  4tgl  5tgl | -  10.15  10.15  - | -  10.5  10.5  - |
| *Rhizopus niveus* | 1lgy | 10.15 | 10.5 |
| ***Rhizopus oryzae*^#^** | **1tic** | **-** | **-** |
| *Serratia marcescens* | 2qua  2qub | 9.94  9.87 | 10.5  10.5 |
| *Staphylococcus hyicus* | 2hih | 9.8 | 10.5 |
| *Sus scrofa* | 1eth | 8.96 | 12.55 |
| *Thermomyces lanuginosus* | 1dt3  1dt5  1dte  1du4  1ein  1gt6  1tib | 10.08  9.87  10.22  9.94  9.66  10.22  10.29 | 10.43  10.36  10.5  10.5  10.5  10.5  10.5 |
| Arithmetic mean (average lysine ε-amino pK_a_) |  | **9.82** | **10.51** |
|  |  | **10.17** | |

^↓^: lipases that were used in this study are highlighted in bold; ^†^: structural coordinates were obtained from the Brookhaven protein structure database (www.pdb.org); ^‡^: lowest pK_a_-value of solvent accessible lysine ε-amino groups calculated for the respective lipase; ^$^ highest pK_a_-value of solvent accessible lysine ε-amino groups calculated for the respective lipase. All pK_a_-values were calculated using the PROPKA web interface (<http://propka.ki.ku.dk/~drogers/>) [1]; * The three-dimensional structure of BSL-B was modelled based on the 3D structure of BSL-A (1isp). For this homology model the PROPKA web interface calculated a pK_a_ min of 9.87 and a pK_a_ max of 10.5; ^#^ no pK_a_-values could be calculated since the respective lipase structures contain only C_α_-atoms but no information about amino acid side chain atoms. For *R. oryzae* lipase (1tic) the calculated pK_a_-values of the lipase from *R. niveus* (1lgy) can be used, since both enzymes only differ by one substitution (M1A) in the prosequence [2].

[1] Li, H., Robertson, A. D., and Jensen, J. H. (2005) **Very fast empirical prediction and interpretation of protein pK_a_ values,** *Proteins: Struct, Funct, Bioinf* *61*, 704-721.

[2] Di Lorenzo, M., Hidalgo, A., Molina, R., Hermoso, J. A., Pirozzi, D., and Bornscheuer, U. T. (2007) **Enhancement of the stability of a prolipase from *Rhizopus oryzae* toward aldehydes by saturation mutagenesis**, *Appl Environ Microbiol* *73*, 7291-7299.
