# Supplementary material for: Mechanism of acetaldehyde-induced deactivation of microbial lipases
Source: BMC Biochem. 2011 Feb 22;12:10. doi: 10.1186/1471-2091-12-10 (PMC3049140; doi:10.1186/1471-2091-12-10)
Supplement: Additional file 2 — Figure S1: Tributyrine plate assay of BSL-B wild type and BSL-B point variants. Tributyrine plate assay of BSL-B wild type as well as BSL-B point variants in which each lysine residue is substituted by alanine and arginine, respectively. -: E. coli BL21(DE3) carrying the empty vector pET19b. WT: E. coli BL21(DE3) expressing BSL-B wild type enzyme (pET19b + lipB). K X A/R: E. coli BL21(DE3) expressing BSL-B in which the lysine residue (K) at position X is substituted with alanine (A) or arginine (R). [file 1471-2091-12-10-S2.DOC]

**Additional File 2**

**Mechanism of acetaldehyde-induced deactivation of microbial lipases**

**Benjamin Franken, Thorsten Eggert, Karl E. Jaeger, Martina Pohl**

**Figure S1: Tributyrine plate assay of BSL-B wild type and BSL-B point variants**

| - | WT | K25A | K37A | K46A | K66A | K71A | K72A | K90A | K97A | K124A |
| --- | --- | --- | --- | --- | --- | --- | --- | --- | --- | --- |
| 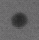 | 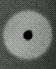 | 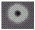 | 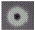 | 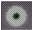 | 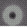 | 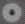 | 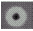 | 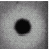 | 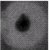 | 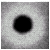 |
| K172A | K25R | K37R | K46R | K66R | K71R | K72R | K90R | K97R | K124R | K172R |
| 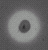 | 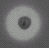 | 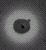 | 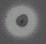 | 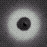 | 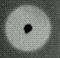 | 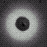 | 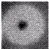 | 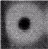 | 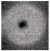 | 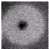 |

Tributyrine plate assay of BSL-B wild type as well as BSL-B point variants in which each lysine residue is substituted by alanine and arginine, respectively. -: *E. coli* BL21(DE3) carrying the empty vector pET19b. WT: *E. coli* BL21(DE3) expressing BSL-B wild type enzyme (pET19b + *lip*B). K X A/R: *E. coli* BL21(DE3) expressing BSL-B in which the lysine residue (K) at position X is substituted with alanine (A) or arginine (R).
