# Supplementary material for: Mechanism of acetaldehyde-induced deactivation of microbial lipases
Source: BMC Biochem. 2011 Feb 22;12:10. doi: 10.1186/1471-2091-12-10 (PMC3049140; doi:10.1186/1471-2091-12-10)
Supplement: Additional file 3 — Table S2: Periodically measured DLS data of differently concentrated BSL-B samples. [file 1471-2091-12-10-S3.DOC]

**Additional File 3**

**Mechanism of acetaldehyde-induced deactivation of microbial lipases**

**Benjamin Franken, Thorsten Eggert, Karl E. Jaeger, Martina Pohl**

**Table S2:** Periodically measured DLS data of differently concentrated BSL-B samples.

|  | **hydrodynamic radii [nm]** | | | **mass fraction [%]** | | | **polydispersities [%]** | | |
| --- | --- | --- | --- | --- | --- | --- | --- | --- | --- |
| **time t [h]** | **100 µg mL^-1^** | | | **250 µg mL^-1^** | | | **500 µg mL^-1^** | | |
| **0** | **3,7** | **100** | **16,1** | **3,4** | **100** | **17,5** | **3,7** | **100** | **26,3** |
| **1** | **3,8** | **100** | **18,8** | **4,2** | **100** | **25,1** | **6,2** | **100** | **28,0** |
| **2** | **3,9** | **100** | **24,8** | **4,3** | **100** | **32,0** | **6,9** | **100** | **36,0** |
| **3** | **3,7**  **9,9** | **97**  **3** | **28,4**  **30,3** | **4,5** | **100** | **41,1** | **7,1** | **100** | **37,8** |
| **4** | **3,0**  **16,5** | **98**  **1,9** | **15,8**  **36,2** | **3,8** | **100** | **33,1** | **6,6** | **100** | **33,7** |
| **5** | **3,9** | **100** | **35,2** | **6,1** | **100** | **44,1** | **9,3** | **100** | **36,8** |
| **6** | **3,8**  **9,2** | **96,2**  **3,2** | **29,6**  **17,7** | **3,0**  **12,3** | **83,7**  **16,1** | **16,5**  **39,6** | **11,3** | **100** | **37,7** |
| **24** | **4,4**  **28,7** | **97,4**  **2,5** | **13,1**  **46,5** | **22,8** | **100** | **67,7** | **20,2**  **374,9**  **9356** | **74,3**  **2,1**  **23,6** | **44,4**  **74,5**  **71,4** |

Due to the detection limit of the DLS apparatus samples with lipase concentrations < 0.1 mg/mL, which were used in the previous experiments could not be analyzed. Based on the periodically measured polydispersities and hydrodynamic radii, spontaneous formation of soluble aggregates could be confirmed. Aggregation velocity directly correlates with the enzyme concentration. At 0.1 mg/mL aggregation during the first 2 h could only be detected by increasing polydispersities, whereas at concentrations between 0.25 and 0.5 mg/mL polydispersities as well as hydrodynamic radii increased, with the 0.5 mg/mL sample showing the fastest increase of both values. In this context the marginal aggregation behaviour at 0.1 mg/mL can be traced back to the fact that this concentration coincides with the transition concentration between the concentration-dependent and concentration-independent area (Figure 3).
