# Supplementary material for: Mechanism of acetaldehyde-induced deactivation of microbial lipases
Source: BMC Biochem. 2011 Feb 22;12:10. doi: 10.1186/1471-2091-12-10 (PMC3049140; doi:10.1186/1471-2091-12-10)
Supplement: Additional file 4 — Table S3: PCR Primers QuikChange-PCR-primer sequences for site-directed mutagenesis of each lysine residue in BSL-B for alanine and arginine, respectively. Each primer pair (e.g. lipB-K25R-fw und lipB-K25A-fw) differs only in the mutagenesis sequence (bold and underlined). [file 1471-2091-12-10-S4.DOC]

**Additional File 4**

**Mechanism of acetaldehyde-induced deactivation of microbial lipases**

**Benjamin Franken, Thorsten Eggert, Karl E. Jaeger, Martina Pohl**

**Table S2: PCR Primers** QuikChange-PCR-primer sequences for site-directed mutagenesis of each lysine residue in BSL-B for alanine and arginine, respectively. Each primer pair (e.g. *lip*B-K25R-fw und *lip*B-K25A-fw) differs only in the mutagenesis sequence (bold and underlined).

| PCR-primer | nucleotide sequence (5‘ → 3‘) |
| --- | --- |
| *lip*B-K25R-fw  *lip*B-K25A-fw | CAA CTT TTT CGC TAT T**CG C**AA CTA CTT AAT TTC T  **GC A** |
| *lip*B-K25R-rev  *lip*B-K25A-rev | AGA AAT TAA GTA GTT **GCG** AAT AGC GAA AAA GTT G  **TGC** |
| *lip*B-K37R-fw  *lip*B-K37A-fw | GGC TGG CAA AGC AAC **CGC** CTG TAC GCA ATT GAT  **GCA** |
| *lip*B-K37R-rev  *lip*B-K37A-rev | ATC AAT TGC GTA CAG **GCG** GTT GCT TTG CCA GCC  **TGC** |
| *lip*B-K46R-fw  *lip*B-K46A-fw | CAA TTG ATT TTT ATG AT**C GC**A CAG GAA ACA ACC TA  **G CA** |
| *lip*B-K46R-rev  *lip*B-K46A-rev | TAG GTT GTT TCC TGT **GCG** ATC ATA AAA ATC AAT TG  **TGC** |
| *lip*B-K66R-fw  *lip*B-K66A-fw | GTT GAC CGT GTT TTA **CGC** GAG ACT GGG GCA AAA  **GCA** |
| *lip*B-K66R-rev  *lip*B-K66A-rev | TTT TGC CCC AGT CTC **GCG** TAA AAC ACG GTC AAC  **TGC** |
| *lip*B-K71R-fw  *lip*B-K71A-fw | GTT TTA AAA GAG ACT GGG GCA **CGC** AAA GTA GAT ATT GTG  **GCA** |
| *lip*B-K71R-rev  *lip*B-K71A-rev | CAC AAT ATC TAC TTT **GCG** TGC CCC AGT CTC TTT TAA AAC  **TGC** |
| *lip*B-K72R-fw  *lip*B-K72A-fw | GAG ACT GGG GCA AAA **CGC** GTA GAT ATT GTG GCT  **GCA** |
| *lip*B-K72R-rev  *lip*B-K72A-rev | AGC CAC AAT ATC TAC **GCG** TTT TGC CCC AGT CTC  **TGC** |
| *lip*B-K90R-fw  *lip*B-K90A-fw | CAA TAC GCT GTA CTA TAT T**CG C**TA TTT AGG CGG GGG C  **GC A** |
| *lip*B-K90R-rev  *lip*B-K90A-rev | GCC CCC GCC TAA ATA **GCG** AAT ATA GTA CAG CGT ATT G  **TGC** |
| *lip*B-K97R-fw  *lip*B-K97A-fw | TTA GGC GGG GGC AAT **GCG** ATT CAA AAT GTC G  **TGC** |
| *lip*B-K97R-rev  *lip*B-K97A-rev | CGA CAT TTT GAA T**CG C**AT TGC CCC CGC CTA A  **GC A** |
| *lip*B-K124R-fw  *lip*B-K124A-fw | ACA GAC CCT AAT CAA **GCG** ATC CTC TAT ACA TC  **TGC** |
| *lip*B-K124R-rev  *lip*B-K124A-rev | GAT GTA TAG AGG AT**C GC**T TGA TTA GGG TCT GT  **G CA** |
| *lip*B-K172R-fw  *lip*B-K172A-fw | TGA ACG GCT ATA TCA **GCG** AAG GGC TGA ATG G  **TGC** |
| *lip*B-K172R-rev  *lip*B-K172A-rev | CCA TTC AGC CCT T**CG C**TG ATA TAG CCG TTC A  **GC A** |
